# Heterozygosity is linked to the costs of immunity in nestling great tits (*Parus major*)

**DOI:** 10.1002/ece3.854

**Published:** 2013-11-05

**Authors:** Beatrice Voegeli, Verena Saladin, Michèle Wegmann, Heinz Richner

**Affiliations:** Evolutionary Ecology Lab, Institute of Ecology and Evolution, University of BernBaltzerstrasse 6, 3012, Bern, Switzerland

**Keywords:** Heterozygosity–fitness correlation, immunity, marker functionality, parasites

## Abstract

There is growing evidence that heterozygosity–fitness correlations (HFCs) are more pronounced under harsh conditions. Empirical evidence suggests a mediating effect of parasite infestation on the occurrence of HFCs. Parasites have the potential to mediate HFCs not only by generally causing high stress levels but also by inducing resource allocation tradeoffs between the necessary investments in immunity and other costly functions. To investigate the relative importance of these two mechanisms, we manipulated growth conditions of great tit nestlings by brood size manipulation, which modifies nestling competition, and simultaneously infested broods with ectoparasites. We investigated under which treatment conditions HFCs arise and, second, whether heterozygosity is linked to tradeoff decisions between immunity and growth. We classified microsatellites as neutral or presumed functional and analyzed these effects separately. Neutral heterozygosity was positively related to the immune response to a novel antigen in parasite-free nests, but not in infested nests. For nestlings with lower heterozygosity levels, the investments in immunity under parasite pressure came at the expenses of reduced feather growth, survival, and female body condition. Functional heterozygosity was negatively related to nestling immune response regardless of the growth conditions. These contrasting effects of functional and neutral markers might indicate different underlying mechanisms causing the HFCs. Our results confirm the importance of considering marker functionality in HFC studies and indicate that parasites mediate HFCs by influencing the costs of immune defense rather than by a general increase in environmental harshness levels.

## Introduction

Associations between individual genetic diversity and fitness-related traits are commonly known as heterozygosity–fitness correlations (HFCs) and have been intensively studied in the last decades (reviewed in, e.g., Hansson and Westerberg 2002; Kempenaers 2007). The underlying assumption for these studies is that heterozygosity is generally beneficial for individuals, mainly because high heterozygosity levels decrease the risk of expressing recessive deleterious alleles (Keller and Waller 2002).

Inbreeding is associated with a decline in heterozygosity levels across the genome and has traditionally been used to explain HFCs, with positive correlations indicating inbreeding depression and negative correlations indicating outbreeding depression. For HFCs to capture information on inbreeding levels, it is necessary that heterozygosity correlates across loci (identity disequilibrium (ID) Szulkin et al. 2010). Under this scenario, HFCs arise by genome-wide effects of heterozygosity, which is commonly referred to as the “general effect hypothesis” (David 1998; Hansson and Westerberg 2002). However, it is strongly debated in the HFC literature whether heterozygosity measured across a set of genetic markers could reflect genome-wide heterozygosity and therefore inbreeding levels (Balloux et al. 2004; Forstmeier et al. 2012). Alternatively, marker heterozygosity might reflect heterozygosity states at closely linked loci only (Balloux et al. 2004). Hence, the “local effect hypothesis” states that HFCs occur due to linkage disequilibrium between genetic markers and loci under selection (Hansson and Westerberg 2002). As the effect of the small number of markers linked to loci under selection will be diluted by the higher number of unlinked loci, local effects are very difficult to detect (Szulkin et al. 2010). The “direct effect hypothesis” holds that the scored markers *per se* have an effect, that is, are functional. As microsatellite markers have traditionally been assumed to be evolutionarily neutral (Queller et al. 1993; Jarne and Lagoda 1996), this hypothesis has gained less attention. However, there is growing evidence on the functional importance of markers located within expressed regions of the genome and within genes (Li et al. 2004). The functionality of markers, that is, whether they are neutral or presumed functional due to their location in expressed genome areas, is linked to the mechanisms of how HFCs can arise. Neutral markers can cause HFCs either by general effects or local effects, if they happen to be closely linked to functional loci. Direct effects, however, can only be caused by functional markers.

Many studies investigated HFCs and reported associations with important life-history traits (reviewed, e.g., in Kempenaers 2007), but high variation in correlational strength was demonstrated between populations and years (Coltman and Slate 2003). The expression and the magnitude of HFCs as well as the strength of inbreeding depression have been suggested to be condition dependent (Balloux et al. 2004; Armbruster and Reed 2005; Chapman et al. 2009) with more pronounced correlations arising under harsh environmental conditions (Lesbarreres et al. 2005; Da Silva et al. 2006; Marr et al. 2006; Fox and Reed 2011). Also sex-specific effects of heterozygosity and inbreeding have been reported previously, with differences between the sexes in the direction and/or strength of the occurring correlations (Coulson et al. 1999; Foerster et al. 2003; Reid et al. 2007; Olano-Marin et al. 2011), possibly explained by sex-specific differences in mortality, growth strategies, and/or resource allocation tradeoffs.

Previous studies also reported a mediating effect of parasite pressure on the occurrence of HFCs (Coltman et al. 1999; Voegeli et al. 2012). Given that parasite abundance can vary between populations and years (Krasnov and Lareschi 2010; Gomez-Flores et al. 2011) and often depends on weather conditions (Merino and Potti 1996), variation in parasite abundance may underlie variation in the occurrence of HFCs under many conditions. Facing parasite infestation, hosts should develop a highly functional immune system to fight and control parasitic infestations as parasites decrease host condition and survival (e.g., Lehmann 1993; Richner et al. 1993). However, the development and maintenance of a competent immune system and the mounting of an immune response are energetically costly, and the limited availability of resources results in tradeoffs with other costly functions (Sheldon and Verhulst 1996; Lochmiller and Deerenberg 2000). A number of studies investigated the investment in the immune system on the extent of growth in juvenile birds and revealed costs of immunity in terms of reduced growth (Saino et al. 1998; Soler et al. 2003; Brommer 2004) and increased mortality (Pitala et al. 2010). Given that immunocompetence is often associated with heterozygosity (Reid et al. 2007; Fossoy et al. 2009), individual heterozygosity may influence the costs derived from mounting an immune response.

In this study, we investigated the relative importance of two possible mediators on the occurrence of HFCs. We manipulated natural brood size, a treatment that is known to alter nestling competition and begging activity (Neuenschwander et al. 2003). Simultaneously, we infested half of the nests with hen fleas (*Ceratophyllus gallinae*), a naturally occurring nest-based ectoparasite, for which we detected a mediating effect on HFCs in a previous study (Voegeli et al. 2012). We then investigated whether nestling heterozygosity is linked to tradeoff decisions between immunity and growth and how the two treatments reinforce these relationships. Furthermore, we tested whether marker functionality, presumed functional or neutral, results in different heterozygosity relationships with the investigated traits.

## Materials and Methods

### Experimental setup

**Figure 1:**
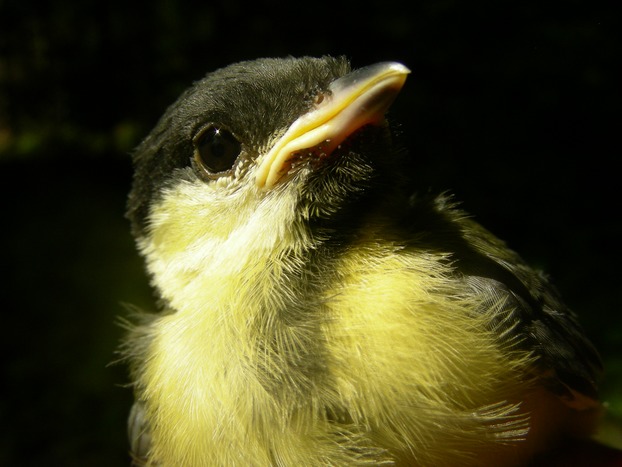
Great tit nestling 15 days after hatching.

The experiment was performed in spring 2011 in a population of great tits (Fig. 1) in a forest near Bern, Switzerland. Three months before the start of the breeding season, we emptied and cleaned all nestboxes in the area. Old nesting material was stored in a climatic chamber at 5°C and was later used to collect hen fleas for the infestation treatment. Nestboxes were regularly visited to determine clutch size, incubation start, and hatching day. We weighed newly hatched nestlings to the nearest 0.01 g and marked them individually by partially removing tuft feathers. The day when the first chick hatched will further be referred to as day 1 for the whole brood. Great tits usually show a hatching spread of up to 3 days (Haftorn 1981). Therefore, we decided to start our treatment when nestlings were 4 days old to ensure that all nestlings would have hatched before.

Brood size manipulation treatment and flea infestation were combined in a 3 × 2 factorial design. To separate genetic and environmental influences on nestling development, broods with identical hatching day (±1 day) were cross-fostered on day 4 by swapping all chicks. We combined the cross-fostering with the brood size manipulation treatment by pairing either broods with the same number of nestlings (unchanged brood size) or broods that differed by two nestlings to create enlarged and reduced broods. We heat-treated nesting material for 3 min using a microwave oven and brushed the nestboxes to remove all remaining parasites (Richner et al. 1993). Each pair of cross-fostered broods was randomly assigned to remain parasite-free or to get infested with 100 fleas, collected from the stored nesting material.

We weighed all nestlings 4 days posthatching and collected blood samples, which were stored in 96% ethanol for genetic analyses. Nestlings were sexed using sexing primers 2917/3088 (Ellegren 1996). Nine days posthatching, nestlings were individually ringed using standard aluminum rings. Nestlings were weighed again when 15 days old, and metatarsus length (±0.1 mm) and the length of the third primary feather (±0.5 mm) were measured. Nestling body condition was then calculated as the residual value of the regression of body mass and tarsus length. Adults were captured on day 12 using a spring trap. Body measurements and blood samples were taken, and blood samples were stored in 96% ethanol for genetic analysis.

### Immune measure

We assessed immune response of nestlings by mimicking a bacterial infection using lipopolysaccharide (LPS; Parmentier et al. 1998), an endotoxin present in the cell walls of Gram-negative bacteria. LPS promotes the release of cytokines and induces an inflammatory response at the injection site (Dunn and Wang 1995). We injected 0.01 mg of LPS (Sigma, Buchs, Switzerland) dissolved in 0.02 mL of phosphate-buffered saline (PBS) into the right wing web of 15-day-old nestlings. Great tit nestlings have been previously shown to have the strongest swelling response 24 h after injection (Berthouly et al. 2008). We therefore measured the thickness of the injected patagium prior to and 24 h after injection using a constant-tension dial micrometer (Mitotuyo, Type 2046S). Each measurement was taken three times, and the strength of the swelling response was calculated as the difference between the mean value before and after the injection. All nestlings from a brood were handled by the same person on both days.

### Genetic analyses

DNA was extracted from blood samples using magnetic beads (MagneSil PMPs, Promega, Dübendorf, Switzerland). We amplified 47 autosomal microsatellite markers by polymerase chain reaction using QIAGEN Multiplex PCR kit (QIAGEN AG, Hombrechtikon, Switzerland) as described in Saladin and Richner (2012).

We divided the set of microsatellite markers into presumed functional and neutral loci using the method described in Olano-Marin et al. (2011). Briefly, we run a BLAST search for all 47 microsatellite sequences using BLASTN (http://blast.ncbi.nlm.nih.gov) against nucleotide collection of zebra finch and chicken. Markers showing homology to avian expressed sequence tags (ESTs) were considered as presumed functional, otherwise as neutral. According to this method, 13 of the microsatellite markers used were identified as presumed functional (Gf4 (Petren 1998), PAT MP 2-14 (Otter et al. 1998), PmaGAn30, PmaGAn31, PmaTAGAn71, PmaTAGAn86 (Saladin et al. 2003), TG01-124, TG03-098, TG05-046, TG06-009, TG08-024, TG12-015 (Dawson et al. 2010), Tgu07 (Slate et al. 2007), and all others were classified as neutral (34 markers). A subset of 11 microsatellite markers was used to analyze nestling paternity status (Saladin et al. 2003). Nestlings were considered as extra-pair offspring if their genotype mismatched their putative father's at two or more loci.

We sampled a total of 1073 nestlings, 116 breeding males, and 119 breeding females from 129 broods across a minimal number of 46 microsatellite markers. Deviations from Hardy–Weinberg equilibrium (HWE) and linkage disequilibrium (LD) were calculated using FSTAT (version 2.9.3; Goudet 1995), using genetic data from adults only to avoid bias due to family structures. None of the markers used showed significant deviation from HWE, and no pair of markers was found to be in LD. We calculated homozygosity by loci (HL) as a measure of individual heterozygosity using Rhh, an extension package for R (Alho et al. 2010). HL gives higher weight to more informative loci by taking into account the allelic variability in each locus (Aparicio et al. 2006). To avoid bias due to family structures, we based calculations of allele frequency on the adult data set only. We used Het_HL_, defined as the difference of 1 – HL, to make high levels of the estimator reflect high levels of heterozygosity. We standardized single-locus heterozygosity (SLH) as explained in Szulkin et al. (2010) to give more weight to more heterozygous loci. We tested for correlations in heterozygosity across loci by calculating (1) heterozygosity–heterozygosity correlations (HHCs; Balloux et al. 2004) with the Rhh extension package for R and (2) the parameter *g*_2_ using RMES, a population genetic freeware detailed in (David et al. 2007).

### Statistical procedures

Nestling swelling response to LPS, feather length, and body condition shortly before fledging were modeled using linear mixed-effect models with restricted maximum-likelihood estimation (REML). Explanatory variables in the starting models were heterozygosity levels of nestlings, nestling sex, flea infestation treatment, and brood size manipulation. We included heterozygosity levels of foster fathers and foster mothers and original brood size as covariates. We controlled for hatching rank by including it as a two-level factor, indicating whether a nestling hatched on the first day (level 1) or later (level 2). When modeling the swelling response to LPS, we additionally included nestling weight on day 15. The random structure consisted of nest identity, in case of the swelling response nested within observer identity, to correct for the nonindependence of siblings. For all models, we eliminated nonsignificant interactions (*α* = 0.1) starting with the highest order interactions and retained all main effects. The 4-factorial interactions were never significant and thus removed from all models. We started instead with the three-way interactions, which included heterozygosity. To interpret significant interactions, we split the models according to factor levels. The fit of the models was verified by checking residuals for normality and homoscedasticity and by plotting residuals against fitted values. Nestling survival was analyzed using a generalized linear mixed model (GLMM) with a binomial error structure and logit link with the add-on package lme4 (Bates et al. 2012). We included the same independent variables as mentioned before. We ran all models separately for functional and neutral heterozygosity levels.

We calculated standard effect sizes by z-transforming the response and independent variable following Nakagawa and Cuthill (2007, 2009). However, as we always had several predictors in the model, this method does provide a “semipartial” correlation, which will always be smaller than a partial correlation (Nakagawa and Cuthill 2009). We used “within-group centering” (Van de Pol and Wright 2009) to separate within- from between-brood effects of nestling heterozygosity. The within-brood effect was assessed by subtracting the brood mean heterozygosity level from each individual nestling heterozygosity estimator. The between-brood effect was simply assessed by the mean values for each brood. Both new predictor variables were then included as fixed effects into the model. Whenever significant within-nest effects were detected, we removed extra-pair nestlings from the data set and rerun the models. However, this procedure never changed the results gained from the models based on the full data set.

We tested for single-locus effects following the approach described in Szulkin et al. (2010). In model 1, the response variable was fitted against Het_HL_ and all other variables retained in the final model, while in model 2, all standardized single-locus heterozygosity measures were included along with the covariates. An *F*-ratio test was used to test whether model 2 explained significantly more variance than model 1. In several of our final models, heterozygosity was part of a significant interaction term with sex or one of the treatments. In this case, and to avoid overparametrization of the model, we split the data according to sex or treatment and tested for SLH separately in the data subsets.

## Results

Heterozygosity levels calculated based on the set of neutral microsatellites and presumed functional markers did not correlate (*r* = −0.023, *P* = 0.73). Presumed functional markers were less diverse compared with neutral markers in terms of allele number (mean number of alleles: functional 5.8 and neutral 15.4) as well as mean observed heterozygosity (functional: 0.44 ± 0.12 and neutral: 0.73 ± 0.07).

Heterozygosity–heterozygosity correlations and *g*_2_ for functional markers were not significantly different from zero (*r*_HHC_ = −0.0003, *P* = 0.82; *g*_2_ = 0.002, *P* = 0.36). For neutral markers, there was a weak trend for positive HHCs (*r*_HHC_ = 0.07, *P* = 0.07), but *g*_2_ did again not differ from zero (*g*_2_ = 0.007, *P* = 0.21).

### Relationship between neutral heterozygosity and fitness measures

**Figure 2:**
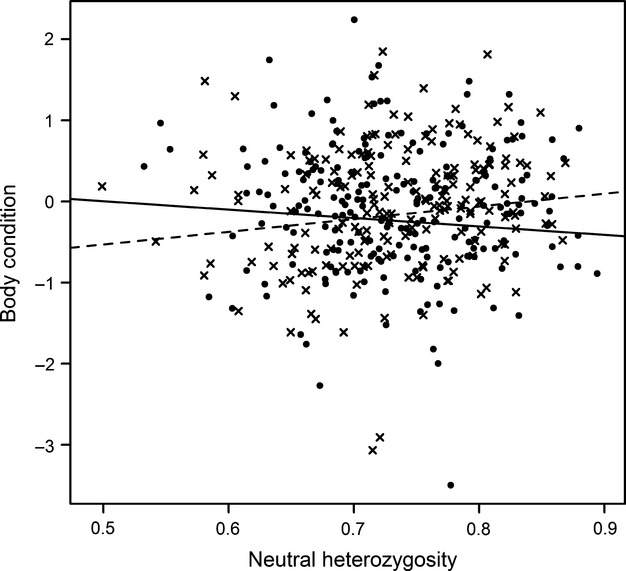
Nestling swelling response to LPS in relation to nestling neutral heterozygosity and parasite treatment. Intercept and slope of the lines are those obtained by the linear mixed-effect model. See text for details. Noninfested broods: black dots and solid line. Parasitized broods: crosses and dashed line.

The relationship between neutral heterozygosity and the swelling response to LPS was influenced by the flea infestation treatment, as indicated by the significant interaction term (Table 1A). In parasite-free control nests, we found a positive relationship between nestling heterozygosity and the swelling response (*F*_1, 285_ = 3.76, *P* = 0.05, *r*_b_ = 0.092), while no significant relationship could be detected in parasitized nests (*F*_1, 295_ = 1.62, *P* = 0.20; Fig. 2).

**Table 1:** Relationship between neutral heterozygosity and nestling fitness traits. (A) Nestling swelling response, (B) feather length, and (C) body condition

Variables	Standardized estimate	Estimate	SE	*F*	df	*P*
(A)						
Intercept		−38.698	20.838	3.449	1, 583	0.064
**Het**_**HLneutral**_	**0.093**	**20.112**	**9.765**	**4.242**	**1, 583**	**0.040**
Het_HLneutral_ foster male	0.034	7.174	15.047	0.227	1, 84	0.635
Het_HLneutral_ foster female	0.097	20.994	16.173	1.685	1, 84	0.198
Sex^a^	−0.048	−1.427	0.881	2.627	1, 583	0.106
Flea infestation^b^	0.535	15.793	10.100	2.445	1, 84	0.122
Enlarged brood^c^	0.092	2.789	2.617	0.956	2, 84	0.389
Reduced brood^c^	−0.029	−0.943	2.722	–	–	–
Hatching rank^d^	0.04	1.268	0.950	1.782	1, 583	0.183
Original brood size	0.011	0.097	0.715	0.018	1, 84	0.893
**Weight**	**0.237**	**1.871**	**0.431**	**18.846**	**1, 583**	**<0.001**
**Het**_**HLneutral**_ * Flea infestation^b^	−**0.759**	−**30.170**	**13.463**	**5.022**	**1, 583**	**0.025**
(B)						
Intercept		45.459	5.172	77.240	1, 628	<0.001
Het_HLneutral_	−0.026	−1.366	1.903	0.515	1, 628	0.473
Het_HLneutral_ foster male	−0.086	−4.403	4.438	0.984	1, 90	0.324
Het_HLneutral_ foster female	−0.061	−3.220	4.743	0.461	1, 90	0.499
Sex^a^	0.032	0.231	0.156	2.207	1, 628	0.138
**Flea infestation**^b^	−**0.691**	−**4.964**	**1.997**	**6.181**	**1, 90**	**0.015**
**Enlarged brood**^c^	−**0.202**	−**1.495**	**0.765**	**3.325**	**2, 90**	**0.040**
**Reduced brood**^c^	**0.062**	**0.480**	**0.786**	**–**	**–**	**–**
**Hatching rank**^d^	−**0.387**	−**2.992**	**0.176**	**287.853**	**1, 628**	**<0.001**
Original brood size	−0.128	−0.272	0.193	1.988	1, 90	0.162
**Het**_**HLneutral**_ * Flea infestation^b^	**0.605**	**5.876**	**2.593**	**5.135**	**1, 628**	**0.024**
(C)						
Intercept		1.070	0.754	2.011	1, 607	0.157
Het_HLneutral_	−0.084	−0.929	0.847	1.204	1, 607	0.273
Het_HLneutral_ foster male	0.013	0.139	0.423	0.108	1, 88	0.743
Het_HLneutral_ foster female	−0.027	−0.299	0.437	0.469	1, 88	0.495
Sex^a^	−0.818	−1.263	0.868	2.117	1, 607	0.146
**Flea infestation**^b^	−**1.176**	−**1.819**	**0.870**	**4.365**	**1, 88**	**0.040**
Enlarged brood^c^	−0.032	−0.051	0.070	0.365	2, 88	0.695
Reduced brood^c^	0.003	0.005	0.078	–	–	–
**Hatching rank**^d^	−**0.226**	−**0.377**	**0.062**	**37.504**	**1, 607**	**<0.001**
Original brood size	−0.057	−0.026	0.019	1.836	1, 88	0.179
Het_HLneutral_ * Sex^a^	0.963	2.013	1.186	2.880	1, 607	0.090
**Het**_**HLneutral**_ * Flea infestation^b^	**1.241**	**2.593**	**1.187**	**4.772**	**1, 607**	**0.029**
Sex * Flea infestation^a^^**,**^^b^	**1.545**	**2.663**	**1.203**	**4.900**	**1, 607**	**0.027**
**Het**_**HLneutral**_ * Sex * Flea infestation^a^^**,**^^b^	−**1.669**	−**3.908**	**1.639**	**5.688**	**1, 607**	**0.017**

SE, standard error.

Results from linear mixed-effect models. All models included nest identity as a random factor to control for the nonindependence among siblings. In the model for the swelling response, the random structure was fitted as nest identity nested within observer. Significant main effects and interactions are shown in bold.

a Relative to female nestlings.

b Relative to noninfested broods.

c Relative to unchanged brood size.

d Relative to first-hatched chicks.

**Figure 3:**
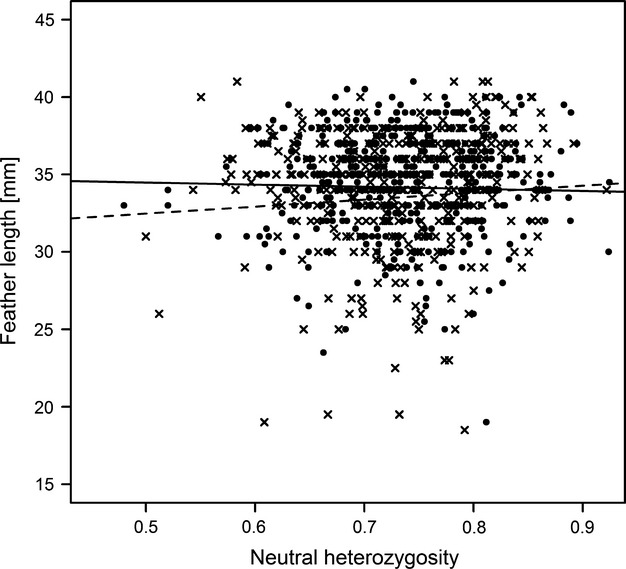
Nestling feather lengths shortly before fledging in relation to nestling neutral heterozygosity and parasite treatment. Intercept and slope of the lines are those obtained by the linear mixed-effect model. See text for details. Noninfested broods: black dots and solid line. Parasitized broods: crosses and dashed line.

Analysis of nestling feather length revealed again a significant interaction between heterozygosity and infestation treatment (Table 1B). For this nestling trait, however, we found a nonsignificant relationship in parasite-free nests (*F*_1,306_ = 0.63, *P* = 0.43) and a positive relationship in parasitized nests (*F*_1,322_ = 6.0, *P* = 0.02, *r*_b_ = 0.086; Fig. 3). Brood enlargement negatively affected nestling feather length (Table 1B).

**Figure 4:**
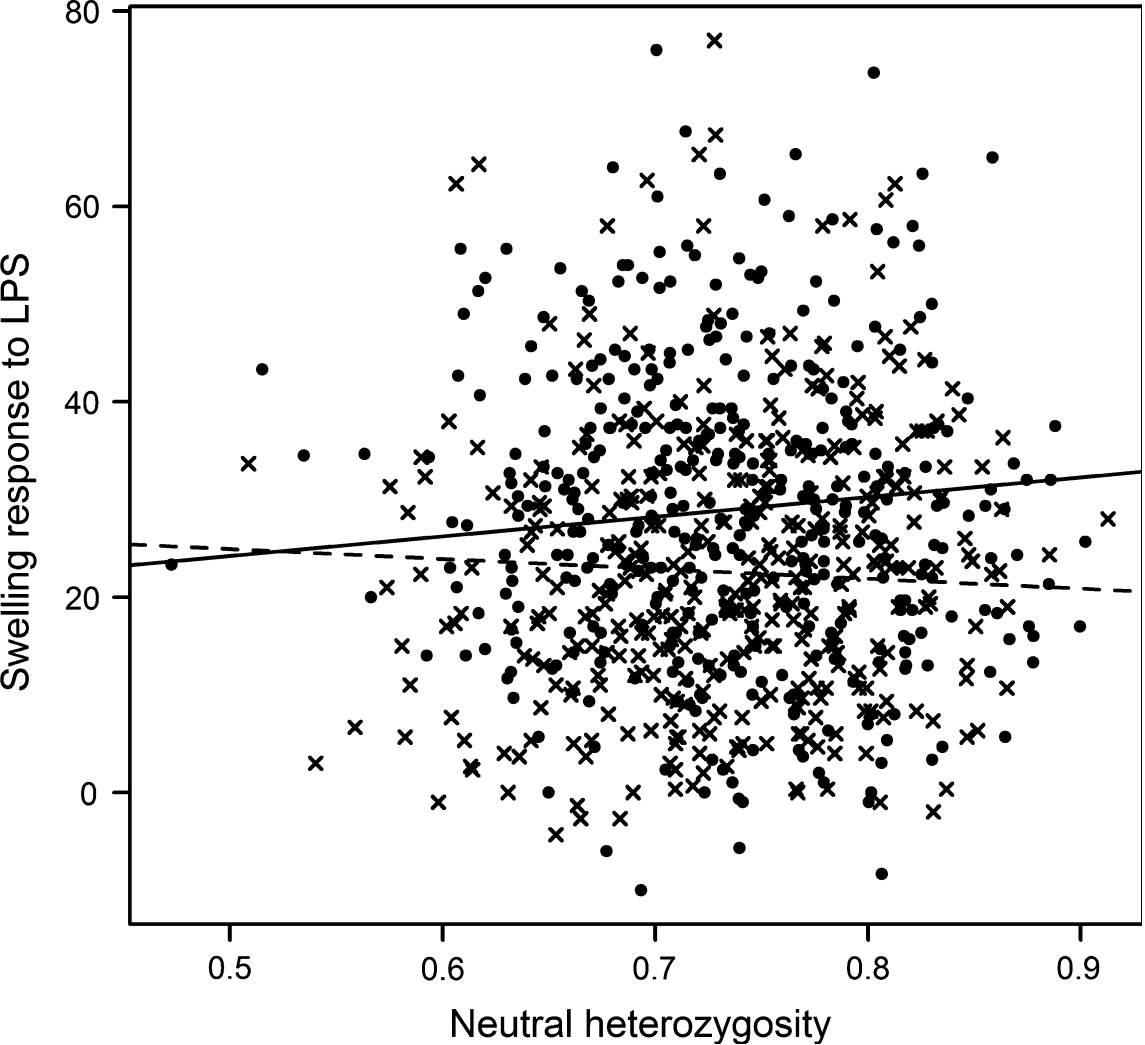
Body conditions of female nestlings in relation to nestling neutral heterozygosity and parasite treatment. Intercept and slope of the lines are those obtained by the linear mixed-effect model. See text for details. Noninfested broods: black dots and solid line. Parasitized broods: crosses and dashed line.

We found a significant interaction between neutral heterozygosity, nestling sex, and flea infestation treatment on nestling body condition (Table 1C). Investigating male and female nestlings separately revealed that female nestlings responded to the infestation treatment differently depending on their heterozygosity levels (Female Het_HL neutral_ * flea infestation: *F*_1,249_ = 4.78, *P* = 0.03). In parasite-free nests, there was no relationship between neutral heterozygosity and body condition of female nestlings (*F*_1,124_ = 1.44, *P* = 0.23), while we found a trend for a positive relationship in parasitized nests (*F*_1,125_ = 3.58, *P* = 0.06, *r*_b_ = 0.138; Fig. 4). In contrast, body condition of male nestlings was affected by neither the interaction of heterozygosity and infestation treatment nor heterozygosity levels alone (Male Het_HL neutral_ * flea infestation: *F*_1,265_ = 0.97, *P* = 0.32; Male Het_HL neutral_: *F*_1,266_ = 2.27, *P* = 0.13).

**Figure 5:**
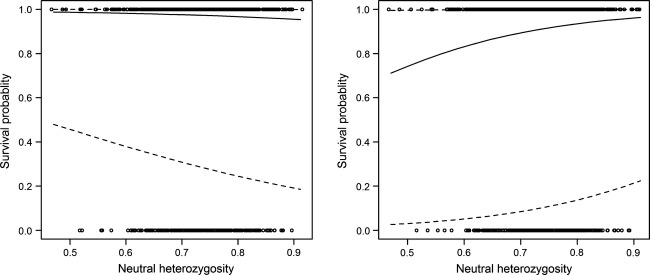
Nestling survival probability in relation to nestling neutral heterozygosity and parasite treatment.

Finally, we found that parasite infestation negatively affected nestling survival and that this negative effect depended again on nestling heterozygosity (Table 2). In parasite-free nests, survival probability was not linked to heterozygosity levels (*z* = −0.91, *P* = 0.36), while in flea-infested nests, the chance of survival was significantly higher for more heterozygous nestlings (*z* = 1.97, *P* = 0.05, *r*_b_ ***=***0.22; Fig. 5). Brood size manipulation did not influence nestling survival, and chicks that hatched later than their siblings had a much lower chance to survive to fledging (Table 2).

**Table 2:** Relationship between nestling heterozygosity and nestling survival probability

Marker	Variables	Standardized estimate	Estimate	SE	*z*	*P*
Neutral	Intercept		11.585	6.991	1.657	0.098
	Het_HLneutral_	−0.735	−3.724	4.123	−0.903	0.366
	Het_HLneutral_ foster male	1.287	6.530	5.622	1.162	0.245
	Het_HLneutral_ foster female	−0.849	−4.474	5.799	−0.771	0.440
	Sex^a^	−0.075	−0.053	0.298	−0.177	0.860
	**Flea infestation**^b^	−**11.542**	−**8.158**	**3.808**	−**2.143**	**0.032**
	Enlarged brood^c^	−1.135	−0.825	0.893	−0.924	0.356
	Reduced brood^c^	1.738	1.332	1.024	1.300	0.194
	**Hatching rank**^d^	−**3.212**	−**2.375**	**0.339**	−**7.010**	**<0.001**
	**Original brood size**	−**2.774**	−**0.592**	**0.294**	−**2.017**	**0.044**
	**Het**_**HLneutral**_ * Flea infestation^b^	**9.83**	**9.419**	**5.035**	**1.871**	**0.061**
Functional	Intercept		10.771	3.526	3.055	0.002
	Het_HLfunctional_	0.114	0.347	1.483	0.234	0.815
	Het_HLfunctional_ foster male	0.833	2.276	2.764	0.824	0.410
	Het_HLfunctional_ foster female	−1.171	−2.804	2.289	−1.225	0.221
	Sex^a^	−0.057	−0.040	0.294	−0.137	0.891
	Flea infestation^b^	−1.923	−1.359	0.741	−1.835	0.066
	Enlarged brood^c^	−1.128	−0.820	0.855	−0.960	0.337
	Reduced brood^c^	1.494	1.145	1.038	1.102	0.270
	**Hatching rank**^d^	−**3.236**	−**2.393**	**0.338**	−**7.086**	**<0.001**
	**Original brood size**	−**2.691**	−**0.575**	**0.291**	−**1.972**	**0.049**

SE, standard error.

Results from generalized linear mixed-effect model with nest identity as a random factor and binomial family structure. Significant main effects and interactions are shown in bold.

a Relative to female nestlings.

b Relative to noninfested broods.

c Relative to unchanged brood size.

d Relative to first-hatched chicks.

### Relationship between functional heterozygosity and fitness measures

Nestling functional heterozygosity was negatively related to the swelling response under all treatments (Table 3A). Neither feather length, body condition (Table 3B,C) nor survival probability (Table 2) were linked to functional heterozygosity levels.

**Table 3:** Relationship between functional heterozygosity and nestling fitness traits. (A) Nestling swelling response, (B) feather length, and (C) body condition

Variables	Standardized estimate	Estimate	SE	*F*	df	*P*
(A)						
Intercept		7.794	13.116	0.353	1, 584	0.553
**Het**_**HLfunctional**_	−**0.068**	−**8.569**	**4.131**	**4.303**	**1, 584**	**0.039**
Het_HLfunctional_ foster male	0.05	5.645	8.100	0.486	1, 84	0.488
Het_HLfunctional_ foster female	−0.122	−12.865	7.145	3.242	1, 84	0.075
Sex^a^	−0.042	−1.231	0.881	1.952	1, 584	0.163
**Flea infestation**^b^	−**0.209**	−**6.162**	**2.076**	**8.811**	**1, 84**	**0.004**
Enlarged brood^c^	0.059	1.776	2.486	1.332	2, 84	0.270
Reduced brood^c^	−0.087	−2.828	2.713	–	–	–
Hatching rank^d^	0.036	1.136	0.952	1.426	1, 584	0.233
Original brood size	0.017	0.153	0.696	0.048	1, 84	0.826
**Weight**	**0.227**	**1.791**	**0.428**	**17.526**	**1, 584**	**<0.001**
(B)						
Intercept		36.830	2.580	203.848	1, 629	<0.001
Het_HLfunctional_	−0.006	−0.190	0.804	0.056	1, 629	0.813
Het_HLfunctional_ foster male	0.115	3.225	2.463	1.715	1, 90	0.194
Het_HLfunctional_ foster female	−0.037	−0.937	2.096	0.200	1, 90	0.656
Sex^a^	0.031	0.226	0.157	2.089	1, 629	0.149
Flea infestation^b^	−0.103	−0.741	0.618	1.438	1, 90	0.234
Enlarged brood^c^	−0.179	−1.319	0.744	2.401	2, 90	0.096
Reduced brood^c^	0.04	0.309	0.805	–	–	–
**Hatching rank**^d^	−**0.386**	−**2.988**	**0.177**	**284.336**	**1, 629**	**<0.001**
Original brood size	−0.099	−0.210	0.194	1.178	1, 90	0.281
(C)						
Intercept		0.320	0.266	1.450	1, 611	0.229
Het_HLfunctional_	−0.057	−0.377	0.245	2.360	1, 611	0.125
Het_HLfunctional_ foster male	0.041	0.245	0.230	1.130	1, 88	0.291
Het_HLfunctional_ foster female	−0.01	−0.055	0.203	0.075	1, 88	0.785
Sex^a^	0.067	0.103	0.057	3.282	1, 611	0.071
Flea infestation^b^	−0.015	−0.023	0.058	0.158	1, 88	0.692
Enlarged brood^c^	−0.026	−0.041	0.068	0.185	2, 88	0.832
Reduced brood^c^	−0.008	−0.014	0.080	–	–	–
**Hatching rank**^d^	−**0.227**	−**0.378**	**0.062**	**37.126**	**1, 611**	**<0.001**
Original brood size	−0.039	−0.017	0.019	0.858	1, 88	0.357

SE, standard error.

Results from linear mixed-effect models. Models included nest identity as a random factor to control for the nonindependence among siblings. In the model for the swelling response, the random structure was fitted as nest identity nested within observer. Significant main effects and interactions are shown in bold.

a Relative to female nestlings.

b Relative to noninfested broods.

c Relative to unchanged brood size.

d Relative to first-hatched chicks.

### Within-group centering and SLH

The relationships between heterozygosity levels and the nestling swelling response, feather length, and body condition could be attributed to effects of heterozygosity within broods, as indicated by the significant within-nest variable in the centered models (Table 4). The relationship between neutral heterozygosity and swelling response showed an additional between-brood component, which differed in the sign from the within-brood component (Table 4). Closer investigation of the among-brood component revealed a significant positive relationship between mean brood heterozygosity and mean swelling response for infested broods, and no significant relationship for parasite-free control broods (infested broods: estimate ±SE = 100.13 ± 39.3, *F*_1,345_ = 6.48, *P* = 0.02; parasite-free broods: estimate ±SE = −57.98 ± 33.65, *F*_1,335_ = 2.9, *P* = 0.09). Testing for single-locus effects following the procedure described in (Szulkin et al. 2010) revealed no evidence for effects of SLH in any of the traits analyzed (Table 5).

**Table 4:** Within- and between-brood effects derived from within-group centering of traits, for which HFCs were significant (see Tables 1–3)

Marker set	Trait	Variable	Estimate	SE	Test statistics	*P*
Neutral	Swelling response	Within * flea infestation	−**43.391**	**13.894**	***F*** **= 9.753**	**0.002**
		Between * flea infestation	**177.913**	**51.368**	***F*** **= 11.996**	**0.001**
	Feather length	Within * flea infestation	**6.311**	**2.633**	***F*** **= 5.746**	**0.017**
		Between * flea infestation	−4.218	15.413	*F* = 0.075	0.785
	Body condition	Within * flea infestation * sex	−**4.316**	**2.032**	***F*** **= 4.510**	**0.034**
		Between * flea infestation * sex	−3.802	2.826	*F* = 1.810	0.180
Functional	Swelling response	**Within**	−**8.992**	**4.33**	***F*** **= 4.313**	**0.038**
		between	−4.301	13.759	*F* = 0.098	0.755
Neutral	Survival	Within * flea infestation	8.487	5.163	*z* = 1.644	0.1
		Between * flea infestation	22.515	20.154	*z* = 1.117	0.264

Results from linear mixed-effect models and GLMM in case of nestling survival. All models included the variables retained in the final models reported previously. Models included nest identity as random factor to control for the nonindependence among siblings. In the model for the swelling response, the random structure was fitted as nest identity nested within observer. Significant effects are shown in bold.

**Table 5:** Test for local effects by comparing variance explained from models including Het_HL_ and models including all SLH

Marker set	Trait and data subset	Data subset	*F*-ratio	*P*
Neutral	Swelling response	Non-infested nests (*N* = 335)	1.01	0.46
	Feather length	Parasitized nests (*N* = 373)	0.61	0.96
	Body condition	Females, parasitized nests (*N* = 172)	0.90	0.63
Functional	Swelling response	Complete data (*N* = 707)	1.03	0.42

To avoid overparametrization when fitting SLH of neutral markers, and significant interactions between heterozygosity and sex or between heterozygosity and flea infestation were split into data subsets, and SLH effects were tested separately in these subsets. An *F*-ratio test was used to test whether the SLH model explained significantly more variance than did the original model based on Het_HL_ values.

## Discussion

In this study, we manipulated growth conditions of nestling great tits by infesting broods with hen fleas and simultaneously manipulating the natural brood size and investigated the mediating effect of both treatments on the occurrence of HFCs. We found that nestlings in experimentally enlarged broods had shorter feathers than control nestlings, indicating that brood enlargement deteriorated growth conditions. However, there was no evidence for a mediating effect of brood size manipulation on the occurrence of HFCs, given that the detected effect did not depend on nestling heterozygosity levels. In contrast, the effects of the flea infestation treatment on the investigated traits strongly depended on nestling heterozygosity levels, joining the evidence that parasites are important mediators of HFCs (Coltman et al. 1999; Voegeli et al. 2012).

### Neutral heterozygosity and fitness

In parasite-free control broods, we found no relationship between nestling heterozygosity and growth or survival, but a positive relationship with the swelling response after LPS injection. These results indicate that while all nestlings invested similar amounts of resources into growth and survival, more heterozygous nestlings could additionally invest also into the development of the immune system. Stronger swelling responses to LPS are thought to reflect higher immunocompetence, and therefore, this positive relationship in parasite-free nests is in line with a number of previous studies reporting positive correlations between individual heterozygosity and immunocompetence or parasite resistance (Acevedo-Whitehouse et al. 2003, 2005; Hawley et al. 2005; Fossoy et al. 2009).

In parasitized nests, no relationship between heterozygosity and immune response could be detected. Ectoparasites have been found to take smaller blood meals from hosts with increased immunocompetence (Bize et al. 2008). Consequently, it may be harmful for a nestling to have a lower immune response than its siblings, possibly explaining why under parasite pressure more homozygous nestlings invested similarly into the development of the immune system as their more heterozygous nest mates. Investments into immunity are known to be costly (Sheldon and Verhulst 1996; Lochmiller and Deerenberg 2000), and our results indicate that these costs depend on nestling heterozygosity levels. We found that more heterozygous nestlings were not only more often surviving the nestling period, but they also grew longer feathers, a trait which is likely to be linked to predator avoidance and postfledging survival (Chin et al. 2009). Both results suggest lower costs of immunity for more heterozygous nestlings. For female nestlings, we found a similar heterozygosity-dependent cost of immunity on the extent of body condition, with more heterozygous females being in better condition shortly before fledging. In contrast, no such cost was found for male nestlings. Sex-specific immunity costs have previously been shown (Dubiec et al. 2006) and may represent different investment strategies. Our results indicate that in the presence of parasites, female nestlings favor investments into immunity over body condition, while males balance both traits.

The detected positive relationships between neutral heterozygosity and survival, feather length, and female body condition in parasitized nests may alternatively be explained not as a cost of increased investments in immunity, but as a result of a generally increased harshness level of the environment. The observation that HFCs are stronger in harsh environments, that is, the parasitized nests, are in line with a number of previous studies (Lesbarreres et al. 2005; Halverson et al. 2006; Marr et al. 2006). However, in the present study, we manipulated the harshness of the rearing conditions in two different ways, not only by flea infestation but also by manipulating brood size. Experimentally altered brood sizes have been previously found to influence, for example, nestling immune response (Horak et al. 1999) and body condition (e.g., Sanz and Tinbergen 1999), probably due to increased nestling competition and limited food availability in enlarged broods. Therefore, if the harshness level of the environment alone predicts the occurrence of HFCs, it is difficult to explain the missing mediating effect of brood size manipulation treatment on HFCs in the present study. In contrast, parasite prevalence does not only increase the general stress level individuals are facing, but also forces exposed hosts to raise an immune response. The costs of developing the immune system and raising an immune response are presumed high (Lochmiller and Deerenberg 2000), and our results suggest that these costs strongly vary with individual neutral heterozygosity.

The correlation between neutral heterozygosity and nestling fitness traits could be attributed to within-brood effects. Removal of extra-pair offspring from the analysis did not qualitatively change these results. Therefore, we can conclude that these within-brood effects were caused by full-siblings. As full-siblings share their ancestry and inbreeding history, HFCs within full-sibling designs are commonly interpreted as evidence for local effects (Hansson et al. 2001; Da Silva et al. 2006; Fossoy et al. 2009). However, it has recently been put forward that the existence of HFCs among full-siblings should be interpreted with care, as full-siblings will vary in the proportion of the genome, which is identical by descent due to chance events during Mendelian segregation (Franklin 1977; Forstmeier et al. 2012). This variation may already be sufficient to cause HFCs even among full-siblings. If local or direct effects underlie the detected HFCs, we would expect to detect single-locus heterozygosity (SLH) effects. However, there was no evidence for SLH among neural markers. Alternatively, the detected HFCs may arise due to genome-wide effects of heterozygosity and hence indicate inbreeding depression. We did find a trend for positive heterozygosity–heterozygosity correlations (HHCs) for neutral markers (Balloux et al. 2004), giving some support to the “general effect hypothesis”. The significant among-brood component of the relationship between neutral heterozygosity and nestling swelling response also supports the general effect hypothesis. Mean brood heterozygosity was positively related to the mean swelling response among infested broods only, which gives further evidence for the mediating effect of parasite infestation on HFCs. This positive HFC on the brood level most likely reflects inbreeding depression in our population.

### Comparing presumed functional and neutral heterozygosity

Heterozygosity measured across a set of presumed functional genetic markers was negatively linked to nestling immune response in all treatments. Thus, functional heterozygosity seemed to have a detrimental effect on nestlings and contrasts with the positive relationships found with neutral markers. The interpretation of negative correlations between heterozygosity and fitness depends on the underlying biological mechanism causing the HFCs. Under the assumption that the detected HFCs are caused by genome-wide effects of heterozygosity (David 1998; Hansson and Westerberg 2002), negative HFCs are interpreted as signals for outbreeding depression. Outbreeding depression can result from the breakup of local adaptations or the disruption of epistatic interactions in offspring of parents originating from divergent populations (Lynch 1991), and it has recently been suggested that functional markers are more suited to detect outbreeding depression than neutral markers due to their lower genetic variability and their location within expressed areas of the genome (Szulkin and David 2011). Neither HHC nor the *g*_2_ values (David et al. 2007) of functional markers were significant, giving no evidence for general effects. However, nonsignificant values should not be misinterpreted as disproving general effects, as the effects of a weak inbreeding are more readily detected on the phenotypic level than on the level of a small number of markers (Szulkin et al. 2010). Also, the number of functional markers used in this study was relatively small, probably causing a lack of power when testing for HHCs. If local or direct effects (Hansson and Westerberg 2002) are underlying the HFCs, the direction of the correlation will depend on the nature of the allelic dominance of the loci causing the HFCs. The correlation could be attributed to within-nest effect, but again no significant SLH effects could be detected.

The negative effects of presumed functional markers and positive effects of neutral markers have previously been explained as resulting from local effects for functional markers and genome-wide effects for neutral ones (Olano-Marin et al. 2011; Laine et al. 2012). This explanation may be valid for our data as well, even though the evidence for local effects of functional markers is weak. Given the low number of functional markers used, we may have been limited in detecting further effects of functional heterozygosity on other traits, and further studies with large data sets in number of markers and individuals are needed to improve our understanding of the often detected negative HFCs with functional markers.

To conclude, in the present study, we found strong evidence for a mediating effect of parasites, but not of brood size manipulation on the occurrence of HFCs. This suggests that parasites mediate HFCs not only by a general increase in the harshness level of the environment, but also by forcing individuals to increase investments into immune defense. Neutral heterozygosity was found to reveal heterozygosity-dependent costs of these increased investments in terms of reduced survival, feather length, and female body condition for less heterozygous nestlings.

## Data accessibility

Data for each individual nestling are deposited at Dryad (http://www.datadryad.org).
